# Primary Prevention of Allergic Diseases: The Role of Early Exposure to Cow's Milk Formula

**DOI:** 10.3389/fped.2020.00420

**Published:** 2020-07-28

**Authors:** Carla Mastrorilli, Angelica Santoro, Carlo Caffarelli

**Affiliations:** ^1^UO Pediatria e Pronto Soccorso, Azienda Ospedaliero-Universitaria Consorziale Policlinico, Ospedale Pediatrico Giovanni XXIII, Bari, Italy; ^2^Clinica Pediatrica, Dipartimento Medicina e Chirurgia, Università di Parma, Parma, Italy

**Keywords:** atopy, allergy prevention, breastfeeding, children, cow's milk allergy, atopic dermatitis, food allergy, asthma

## Abstract

The burden of atopic disorders is continuously worsening worldwide, especially in childhood. Therefore, risk factors and preventive measures have been called into question. The age when infants introduce complementary foods, varies greatly according to traditional habits, clinical practice recommendations, and breastfeeding duration. It is still debated the impact of early exposure to cow's milk on the increase of allergic diseases, mainly food allergy, and atopic dermatitis. Many factors may play a role in this potential link, such as genetic variation, parental atopy, infant feeding regimens. Recent evidences suggest that the early introduction of complementary foods (up to 6 months of age), including cow's milk, could prevent the development of food allergies. So, several countries included this new approach into feeding guidelines. Our review will focus on the influence of early exposure to cow's milk formula on the development of allergic diseases. Some trials found that cow's milk supplementation in the first days of life could even increase the development of IgE sensitization and food allergies. Other trials did not show any efficacy on prevention of allergic disorders. Further studies are needed to understand the prospective for allergy prevention related to optimal timing of cow's milk formula introduction.

## Introduction

The burden of atopic disorders is continuously increasing worldwide, especially in the pediatric setting. Therefore, several risk factors and diverse preventive measures have been called into question (1). The age when infants introduce complementary foods varies greatly according to traditional habits, clinical practice recommendations, and breastfeeding duration. Several studies suggest that the timing of introduction of food allergens may be fundamental for the development of allergic diseases, principally food allergy, and atopic dermatitis (2). In particular, the effects of early exposure to cow's milk (CM) are still debated. Many factors may play a role in this potential relation (3–5). In the past, clinical practice guidelines had recommended a delayed exposure to allergenic foods including CM, egg, fish, nuts among children with parental atopy (6, 7) to prevent allergy with poor results (8). The timing of exposure to complementary foods corresponds to the healthy gut colonization, found to be crucial in stimulating allergen tolerance (9). So, it has been hypothesized that allergic disorders can be due to immature immunoregulatory networks and reduced diversity and intensity of microbial exposure (10, 11). Moreover, another suggestion of allergy risk is the dual-barrier hypothesis, theorizing that allergic sensitization is a consequence of cutaneous exposure, and tolerance results from oral exposure to foods (3). So, avoidance of specific foods (e.g., egg or peanuts) can increase the risk of developing allergy, especially in high risk infants (e.g., barrier defects, such as eczema or filaggrin deficiency). Therefore, preventive strategies shifted from avoidance to controlled exposure, suggesting that allergen avoidance may be even harmful for allergy prevention. This has raised the search of an “optimal window” for introduction of complementary foods to prevent allergic disorders. Data suggest to start weaning not before 3-4 months of age, because gut colonization and immune network are not well-established yet (12–14). Updated guidelines nowadays recommend introducing complementary foods from 4 to 6 months of age irrespective of potential food allergenicity and atopic family history (15–21). However, if these guidelines can be recommended to general population or only in high-risk infants it has still to be elucidated (22). Moreover, no current guideline defines the optimal timing of introduction of cow's milk formula (CMF) other than that it can be comprised along with other foods.

Cow's milk allergy (CMA) represents the most common food allergy in infancy with an estimated prevalence of 2–5% (23). Only in 60% of cases CMA is IgE-mediated with symptoms, such as urticaria, wheezing, anaphylaxis, starting within 15–30 min after exposure to CMP, even in low amounts (24, 25). Other cases include food protein–induced enterocolitis syndrome (FPIES) or a mixed IgE-associated or cell-mediated reaction, such as atopic dermatitis (26) and eosinophilic gastroenteritis. The child becomes sensitized to food allergens *in utero*, via breastmilk and by ingestion, skin contact, and inhalation (27). Even if it is unclear whether breastfed infants are less prone to develop atopic dermatitis or food allergy, breastfeeding keeps on being fundamental in infants' diet and it is advised at least until 6 months of age (28).

The aim of the current review is focused on the influence of early exposure to cow's milk proteins (CMP) in the first days of life on the development of allergic diseases. We perform a literature search in PubMed and Cochrane library for English-language studies published during the period 1985–2019 that assessed whether early administration of CMF in the first 3–4 days of life was associated with development of CMA or atopic diseases. Information was obtained from randomized controlled trials (RCT) published in peer-reviewed journals. Trials that compared CMF only with hydrolyzed formula, rice formula, soy formula, or other mammalian milk and studies in preterm infants were excluded. In addition, we discussed selected papers that may be useful for the purpose of this review. We planned to provide a practical approach for introducing CMP in the infant's diet.

## Description of RCT Studies

Five RCT (Table 1) met the inclusion criteria (29–35). In 1988, Lindfors and Enocksson (29) and Lindfors et al. (30) randomly assigned a 216 healthy term infants to CMF feeding (*n* = 112) or breastfeeding (*n* = 104) for the first week of life since their earliest meal (at 6 h of age) and studied the incidence of atopic diseases up to 18 months of age. Children were examined by welfare center's pediatricians who participated in the study at 3, 6, 18 months of age and at the age of 4–6 year. Allergic symptoms were recorded as obvious or probable. Moreover, infants with high risk for atopy were assigned to *single* heredity group (one parent or a sibling with a positive history of allergy) or double heredity group (two first-degree relatives with a positive history of allergy). Serum IgE antibodies to inhalants, pediatric food allergen mix and skin prick tests to egg, cow's milk, and inhalants were performed at 4–6 years of age.

**Table 1:** Characteristics of randomized controlled studies on introduction of cow's milk formula in the first days of life.

**Author, Country**	**Population**	**Inclusion criteria**	**N active group/N control group**	**Cow's milk challenge**	**Age at outcome assessment**	**N active group/N control group with cow's milk allergy**	**N active group/N control group with atopic diseases**	**N active group/N control group with other food allergy**	**Feeding after 3 days**
Lindfors and Enocksson. (29), Sweden	General population	Healthy term infant, birth weight between −1 SD and −2 SD	109 cow's milk formula/98 breast milk	No	18 months	Not reported	18%/33% (*p* < 0.05)	Not reported	Cow's milk formula or breast milk
Lindfors et al. (30), Sweden	General population	Healthy term infant, birth weight between −1 SD and −2 SD	95 cow's milk formula/88 breast milk	No	4–6 years	Not reported	20%/32%	Not reported	Cow's milk formula or breast milk
Juvonen et al. (31), Sweden	General population	All newborns	39 cow's milk formula/53 breast milk	No	3 years	0/1	7%/7%	5.1%/5.6%	Breast milk
De Jong et al. (32), Netherlands	General population	Healthy full-term newborns	758 cow's milk formula/775 placebo	No	2 years	Not reported	11.9%/12.4%	Not reported	Breast milk Cow's milk formula if required
De Jong et al. (33), Netherlands	General population	Healthy full-term newborns	542 cow's milk formula/566 placebo	No	5 years	Not reported	26.3%/25%	Not reported	Breast milk Cow's milk formula if required
Saarinen et al. (34), Finland	General population	Healthy full-term newborns	1,758 cow's milk formula/1,844 human milk	Yes	mean (± SD) 6.7 ± 2.3 months	2.4%/1.7%	Not reported	Not reported	Breast milk Cow's milk formula if required
Urashima et al. (35), Japan	At least one 1-degree relative with current and/or past atopic disease	Healthy full-term newborns	156 or breastfed plus cow's milk formula /156 breastfed plus amino acid–based elemental formula	Open food challenge or a suggestive reaction	2 years	6.6%/0.7%	Not reported	Egg, 19.9%/11.3% Wheat, 4.6%/0.7%	In breastfed plus amino acid–based group, 115 infants had breast milk + cow's milk formula, 39 had breast milk/Elemental formula

Juvonen P et al. randomized 129 infants at birth to human milk (HM), CMF, or highly casein hydrolysate formula during the first 3 days of life (31). Subsequently, infants were exclusively breastfed. Children were clinically examined and serum IgE levels to alpha-lactoglobulin were detected at 2, 4, 8, 12, and 24 months of age. At 1 and 2 years of age, infants underwent skin prick tests to food and inhalant allergens. Food allergy or allergic symptoms were diagnosed by a telephone interview at 3 years of age.

A double-blind RCT exposed 1,533 breastfed newborns to CMF or protein free placebo during the first 3 days of life with the aim of detecting the occurrence of atopic diseases in the first 5 years of life by history, physical examination, and questionnaire (32, 33). Serum specific IgE antibodies to cow's milk, egg and inhalants were measured at 1 and 5 year of age.

Saarinen et al. (34) studied whether supplementary feeding of CMF at maternity hospital would influence the frequency of CMA at about 27 months of age when compared with pasteurized HM among 6,209 unselected full-term newborns. CMA was diagnosed by oral food challenge.

A recent Japanese RCT (35) (Atopy Induced by Breastfeeding or Cow's Milk Formula [ABC] trial) randomized 312 newborns with a family history of atopy to breastfed *plus* aminoacid–based elemental formula group in the first 3 days of life (BF/EF) or breastfed plus cow's milk formula (BF/CMF) from the first day of life to 5 months. Primary outcome of the study was rate of positive IgE antibodies to cow's milk (IgE > 0.35 allergen units [UA]/mL) at 2 years of age. The occurrence of immediate food allergy, including CMA, diagnosed by oral food challenge test, or triggered by food ingestion with positive IgE to the relevant food was also investigated.

## Cow's Milk Allergy/Sensitization

The development of CMA diagnosed by oral food challenge that is the gold standard for ascertaining food allergy, in subjects that were early fed with CMF was investigated by two of RCTs we reviewed (34, 35). They showed that the administration of CMF in the first 2–4 days of life may play a role for the onset of CMA. In infants with atopic family history (35), the incidence of CMA at age 2 was lower among breastfed newborns at risk for atopy supplemented with aminoacid-based formula compared to those supplemented with CMF (CMA: 1/151 in BF/EF group in the first 3 days of life vs. 10/151 in BF/CMF group; RR 0.10; 95%CI, 0.01–0.77). Moreover, at 2 years of age, infants in the BF/EF group were less frequently sensitized to CM (specific IgE levels ≥0.35 UA/ml) compared to infants in the BF/CMF group (16 vs. 32%; relative risk, 0.52; 95%CI, 0.34–0.81). Infants who avoided CMF for 3 days could be then fed with CMF according to maternal preferences, and only 39 infants did not receive CMF until 5 months of age. However, a *post-hoc* analysis did not show any difference for food allergy in this subgroup. On the other hand, Saarinen et al. (34) noted a non-significant increase of the risk of CMA in general population who were fed with CMF for an average of 2 days after birth. CMA occurred in 2.4% of infants who received CMF supplementation at maternity hospital vs. 1.7% of those supplemented with pasteurized HM at maternity hospitals [Odd Ratio (95% CI) 0.70 (0.44–1.12)]. The mean follow-up period was 27 months (range, 18–34 months). Juvonen et al. (31) showed that no child who received CMF during the first 3 days of life developed CMA at 3 years of age while one child who received HM developed atopic eczema to cheese. Regarding sensitization to cow's milk, Lindfors et al. (30) found that at 5 years of age, skin prick test results to cow's milk were positive in 3/80 children who were early fed with CMF and in 0/74 children who did not received infant formula. Finally, de Jong et al. (32, 33) found that at 1 and 5 year of age there was no difference in serum specific IgE levels to cow's milk between infants who early received CMF and those who received placebo. Differences in selected populations and study design may explain different findings among studies (36). Several explanations may be offered for the potential enhanced risk for CM sensitization in subjects with allergic family history who in their initial days of life are exposed to CMF. It is possible that an early occasional ingestion of CMF may initiate sensitization and predispose infants to CMA. Accordingly, a retrospective case-control study by Kelly et al. (37) showed that CMA were seven times more frequent in breastfed infants who received CMF in the first 24 h of life than in exclusively breastfed infants. Infants who were exclusively formula fed were not at increased risk for CMA. Along this line, Katz et al. (38) showed that occurrence of CMA was less likely in infants who were regularly exposed to CMF following discontinuation of exclusive or almost exclusive breast-feeding in the first 15 day of life than those who received CMF later in the first year of life. Moreover, a continuous administration of the allergen during allergen specific immunotherapy has been shown to be effective to induce tolerance to the allergen in question (39). Unfortunately, the studies we reviewed did not assessed this issue. Another possibility is that may be due to immature local immune system and bacterial intestinal colonization (13). Indeed, germ free mice rated with antibiotics or empty of any bacteria colonization have exhibited great susceptibility to anaphylaxis and food allergy (10).

## Onset of Allergy/Sensitization to Other Foods

There is no clear evidence that hypersensitivity to foods other than CM was associated with early administration of CMF in the studies we reviewed. In the ABC trial (35), sensitization rate to egg white or wheat did not increase in infants who had CMF at birth. At 2 years of age, the immediate and anaphylactic types of food allergy independent of food type (i.e., not simply CM but additionally wheat, hen's egg, and others) were more common in the group early exposed to CMF. However, it is unclear what are the results when CMA is excluded and the mechanisms are undetermined. Moreover, Juvonen et al. found that at 3 years of age there was no difference in food allergy rates between CMF group and HM group during the first 3 days of life (3/53 infants in the HM group, 2/39 in the CMF group) (31). The most frequently sensitizing food was hen's egg. Finally, de Jong et al. (32, 33), found no differences in serum specific IgE levels for egg at 1 and 5 year of age between active group and placebo group.

## Allergic Symptoms

For atopic diseases, observational studies have reported discordant results on timing of introduction of CMF and their prevention (40–43). Regarding the studies we reviewed, the judgement is unclear and new studies are warranted. A significant association of atopic diseases with early CMF administration has been reported in one case (29, 30) and no link in two cases (31–33). Lindfors et al. (29, 30) showed a significantly increased frequency of allergic symptoms in the formula-fed infants compared to breast-fed infants at 18 months of age, particularly in children with double atopic heredity. At 5 years of age, findings of the study found a significant lower frequency of allergic symptoms among formula-fed infants compared to controls only in the double positive atopic family history group. Juvonen et al. (31) found that infants who avoided CMF were not more likely to develop allergic symptoms at 3 years of age than those who did not avoid. In the trial by de Jong et al. (32, 33) no significant difference was found in the development of atopic diseases among children exposed or not to CMF, at 1 year of life (10 vs. 9.3%), at 2 year of life (9.6 vs. 10.2) and at 5 years of life (26.3 vs. 25%, relative risk 1.05).

## Individual Allergic Symptoms

Atopic dermatitis was not associated with early administration of CMF in the studies we reviewed Indeed, a Swedish study showed that both at 18 months of age (9 vs. 18%) (29) and at 5 years of age (17 vs. 25%) (30) there was no significant difference in the frequency of atopic eczema between infants fed with CMF or breastfed in the first week of life. This was the case also in subgroups with family history of atopy. Over the first 3 years of life, Juvonen et al. (31) showed that the incidence of atopic eczema was not statistically different in infants fed in the first 3 days of life with HM in comparison with those fed with CMF (3/53 vs. 3/39). The results of a follow-up analysis of the trial by de Jong et al. (33) showed that brief neonatal exposure to CMP was not associated with atopic eczema at 5 years of age.

Timing of CM introduction very early in life did not prevent the development of wheeze. In infants fed with CMF or breastfed in the first days of life, Lindfors et al. did not find any difference in the incidence of wheeze, at 18 months of age (29) and at 5 years of age (30), even in relation to family history of allergy. The incidence of asthma until 3 years of age was not different in infants who received CMF vs. HM during the first 3 days of life (3/53 infants in the HM group, 0/39 in the CMF group) (31). At 5 years of age, the frequency of wheeze during the past 12 months was similar in infants who received CMF and in those who received placebo in the first 3 days of life (33). As well, sensitization to inhalant allergens was not dissimilar between intervention groups. Introduction of CMF in the first days of life compared with breastfeeding did not increase the frequency of urticaria and gastrointestinal symptoms at 18 months of age (29) or at 5 years of age (30).

For rhinoconjunctivitis, CMF intake in the first days of life did not increase the risk of developing rhinoconjunctivitis or positive specific IgE antibodies to seasonal or perennial allergens (30, 33). It is difficult to understand why the risk of rhinoconjunctivitis significantly increased among children with double atopic heredity (30).

## Conclusions

There is a long-standing debate on the link between early introduction of CMF and onset of CMA and allergic diseases that led us to review the results of RCT. Our paper extends findings of a previous review (44) by considering more studies (32, 33, 35). Yuan showed that early introduction of CMF did not have any effect on development of asthma, atopic dermatitis and CMA.

Some data in children with positive family history of atopy might suggest that an early exposure to CM may predispose to the onset of CM sensitization. It is unlikely that an early exposure to CMF prevents the development of allergic diseases or hypersensitivity to foods other than CM. However, several items limit conclusions of our review. There is a paucity of studies on the role of early exposure to CM on the prevention of allergy. We search only RCTs in English language so relevant studies might potentially be excluded. Study designs are heterogeneous, and inconsistent. Some trials are small. Findings of trials are divergent and associated to confounders, such as family history of atopy, number of outcomes, duration of breastfeeding, weaning (45), age at analysis, definition. Amount, dose, frequency and composition of formula supplementation may also affect the results. CM challenge test was performed only in two studies (34, 35). A third arm fed with hydrolyzed milk formula has also been introduced in a minority of studies (31, 34). Another issue is that an RCT study may not mirror the real life since the supplementation is often given when a weight loss is observed or lack of breast milk is perceived and it doesn't necessarily continue as milk comes in.

Much more effort is still needed to understand the prospective for allergy prevention related to early exposure to CMF, the optimal timing of CM introduction, continuous intake of CM over time, and the potential consequences of current strategies on breastfeeding.

## Author Contributions

CM, AS, and CC co-wrote the manuscript and approved the final version. All authors contributed to the article and approved the submitted version.

## Conflict of Interest

The authors declare that the research was conducted in the absence of any commercial or financial relationships that could be construed as a potential conflict of interest.
